# Supplementary material for: Geographical variation in demand, utilization, and outcomes of hospital services for COVID-19 in Brazil: A descriptive serial cross-sectional study
Source: PLoS One. 2021 Sep 30;16(9):e0257643. doi: 10.1371/journal.pone.0257643 (PMC8483366; doi:10.1371/journal.pone.0257643)
Supplement: Attachment — Submitted filename: Response to Reviewers.docx [file pone.0257643.s002.docx]

**PONE-D-21-15377**

**Response to Reviewers**

1. Is the manuscript technically sound, and do the data support the conclusions?

The manuscript must describe a technically sound piece of scientific research with data that supports the conclusions. Experiments must have been conducted rigorously, with appropriate controls, replication, and sample sizes. The conclusions must be drawn appropriately based on the data presented.

Reviewer #1:Yes

2. Has the statistical analysis been performed appropriately and rigorously?

Reviewer #1: Yes

3. Have the authors made all data underlying the findings in their manuscript fully available?

The [PLOS Data policy](http://www.plosone.org/static/policies.action#sharing) requires authors to make all data underlying the findings described in their manuscript fully available without restriction, with rare exception (please refer to the Data Availability Statement in the manuscript PDF file). The data should be provided as part of the manuscript or its supporting information, or deposited to a public repository. For example, in addition to summary statistics, the data points behind means, medians and variance measures should be available. If there are restrictions on publicly sharing data—e.g. participant privacy or use of data from a third party—those must be specified.

Reviewer #1: No

**Our response: All the data used in the work were deposited in a public repository** <https://data.mendeley.com/datasets/2ggr5m5gk4/1>**.**

1. Is the manuscript presented in an intelligible fashion and written in standard English?

PLOS ONE does not copyedit accepted manuscripts, so the language in submitted articles must be clear, correct, and unambiguous. Any typographical or grammatical errors should be corrected at revision, so please note any specific errors here.

Reviewer #1:No

**Our response: We have revised the manuscript accordingly.**

5. Review Comments to the Author

Please use the space provided to explain your answers to the questions above. You may also include additional comments for the author, including concerns about dual publication, research ethics, or publication ethics. (Please upload your review as an attachment if it exceeds 20,000 characters)

Reviewer #1: Dear Colleagues, thank you very much for the opportunity to review your scientific paper which tries to elucidate the geographical differences that exist in Brazil in relation to the COVID-19 pandemic.

However, the paper is not written following strobe guidelines, therefore it is very difficult to understand and read the methodology and the differences given in the scientific paper.

When the changes are made, the paper can be reviewed again without any problem.

**Our response: Thank you for your availability to review our manuscript. We have added the STROBE checklist as a supplemental information, indicating where each item is found in the manuscript. We reorganized the Methods in order to comply more strictly with the guidelines. A few items were not applicable to the type of study proposed. Additionally, we would like to underline that we classify our study as a health services research manuscript, providing a thorough description of the background motivation for the work, our methods and results.**

6. PLOS authors have the option to publish the peer review history of their article ([what does this mean?](https://journals.plos.org/plosone/s/editorial-and-peer-review-process#loc-peer-review-history)). If published, this will include your full peer review and any attached files.
If you choose “no”, your identity will remain anonymous but your review may still be made public.

**Do you want your identity to be public for this peer review?** For information about this choice, including consent withdrawal, please see our [Privacy Policy](https://www.plos.org/privacy-policy).

Reviewer #1: No

[NOTE: If reviewer comments were submitted as an attachment file, they will be attached to this email and accessible via the submission site. Please log into your account, locate the manuscript record, and check for the action link "View Attachments". If this link does not appear, there are no attachment files.]

While revising your submission, please upload your figure files to the Preflight Analysis and Conversion Engine (PACE) digital diagnostic tool, <https://pacev2.apexcovantage.com/>. PACE helps ensure that figures meet PLOS requirements. To use PACE, you must first register as a user. Registration is free. Then, login and navigate to the UPLOAD tab, where you will find detailed instructions on how to use the tool. If you encounter any issues or have any questions when using PACE, please email PLOS at [figures@plos.org](mailto:figures@plos.org). Please note that Supporting Information files do not need this step.

**Done.**
